# Tris{2-meth­oxy-6-[(4-methyl­phen­yl)iminiometh­yl]phenolato-κ^2^ *O*,*O*′}tris­(thio­cyanato-κ*N*)praseodymium(III) monohydrate

**DOI:** 10.1107/S1600536809038823

**Published:** 2009-10-03

**Authors:** Jian-Feng Liu, Jia-Lu Liu, Guo-Liang Zhao

**Affiliations:** a Zhejiang Key Laboratory for Reactive Chemistry on Solid Surfaces, Institute of Physical Chemistry, Zhejiang Normal University, Jinhua, Zhejiang 321004, People’s Republic of China, and, College of Chemistry and Life Science, Zhejiang Normal University, Jinhua 321004, Zhejiang, People’s Republic of China

## Abstract

The asymmetric unit of title compound, [Pr(NCS)_3_(C_15_H_15_NO_2_)_3_]·H_2_O, consists of three Schiff base 2-meth­oxy-6-[(4-methyl­phen­yl)imino­meth­yl]phenol (H*L*) ligands, three independent thio­cyanate anions and an uncoordinated water mol­ecule. The Pr^III^ ion is nine-coordinated. The thio­cyanate anions coordinate to the Pr^III^ ion *via* the N atoms and the three H*L* ligands chelate the Pr^III^ ion *via* the phenoxy and meth­oxy O atoms. The protonated imine N atoms are involved in intra­molecular hydrogen bonds with the phenolate groups.

## Related literature

For related structures, see: Li *et al.* (2008 ▶); Liu *et al.* (2009 ▶); Zhao *et al.* (2007 ▶); Xian *et al.* (2008 ▶). For background to our studies of complexes of Schiff bases derived from *o*-vanillin, see: Zhu *et al.* (2005 ▶).
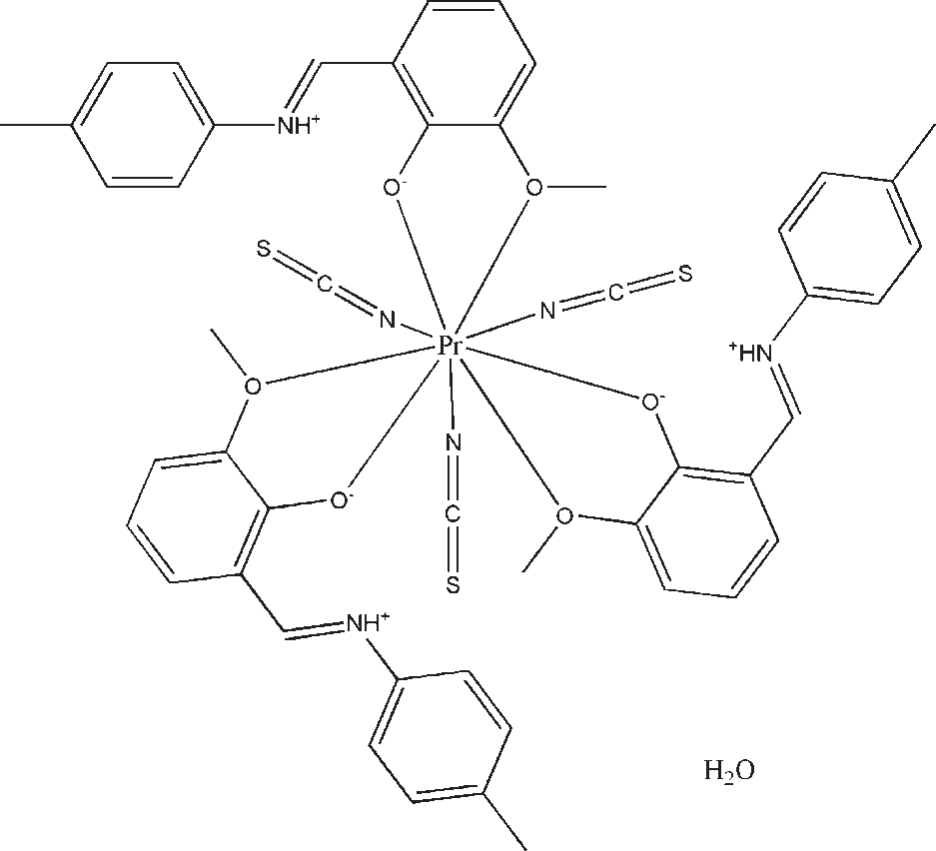


## Experimental

### 

#### Crystal data

[Pr(NCS)_3_(C_15_H_15_NO_2_)_3_]·H_2_O
*M* *_r_* = 1057.01
Monoclinic, 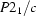

*a* = 16.6770 (6) Å
*b* = 14.2420 (5) Å
*c* = 22.2021 (8) Å
β = 105.810 (2)°
*V* = 5073.8 (3) Å^3^
*Z* = 4
Mo *K*α radiation
μ = 1.14 mm^−1^
*T* = 296 K
0.18 × 0.16 × 0.04 mm

#### Data collection

Bruker APEXII area-detector diffractometer
Absorption correction: multi-scan (*SADABS*; Sheldrick, 1996 ▶) *T*_min_ = 0.819, *T*_max_ = 0.956
39629 measured reflections
8927 independent reflections
6217 reflections with *I* > 2σ(*I*)
*R*_int_ = 0.067

#### Refinement

*R*[*F* ^2^ > 2σ(*F* ^2^)] = 0.039
*wR*(*F* ^2^) = 0.098
*S* = 1.01
8927 reflections
589 parameters
9 restraints
H atoms treated by a mixture of independent and constrained refinement
Δρ_max_ = 0.55 e Å^−3^
Δρ_min_ = −0.40 e Å^−3^

### 

Data collection: *APEX2* (Bruker, 2006 ▶); cell refinement: *SAINT* (Bruker, 2006 ▶); data reduction: *SAINT*; program(s) used to solve structure: *SHELXS97* (Sheldrick, 2008 ▶); program(s) used to refine structure: *SHELXL97* (Sheldrick, 2008 ▶); molecular graphics: *SHELXTL* (Sheldrick, 2008 ▶); software used to prepare material for publication: *SHELXL97*.

## Supplementary Material

Crystal structure: contains datablocks I, global. DOI: 10.1107/S1600536809038823/at2866sup1.cif

Structure factors: contains datablocks I. DOI: 10.1107/S1600536809038823/at2866Isup2.hkl

Additional supplementary materials:  crystallographic information; 3D view; checkCIF report

## Figures and Tables

**Table 1:** Selected bond lengths (Å)

Pr—O5	2.384 (2)
Pr—O1	2.409 (2)
Pr—O3	2.419 (2)
Pr—N4	2.515 (3)
Pr—N6	2.532 (3)
Pr—N5	2.562 (3)
Pr—O4	2.773 (2)
Pr—O2	2.790 (2)
Pr—O6	2.838 (2)

**Table 2:** Hydrogen-bond geometry (Å, °)

*D*—H⋯*A*	*D*—H	H⋯*A*	*D*⋯*A*	*D*—H⋯*A*
N1—H1*A*⋯O1	0.86	1.88	2.572 (3)	137
N2—H2*A*⋯O3	0.86	1.85	2.552 (3)	138
N3—H3*A*⋯O5	0.86	1.88	2.585 (3)	138

**Fig. 1.**
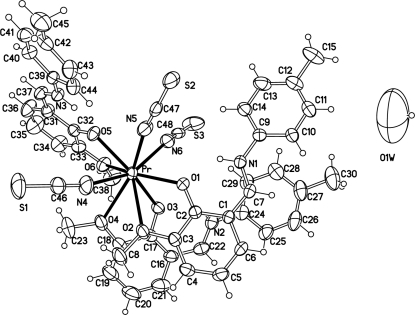
The molecular structure of the title complex, showing the atom-labelling scheme. Displacement ellipsoids are drawn at the 30% probability level.

**Fig. 2.**
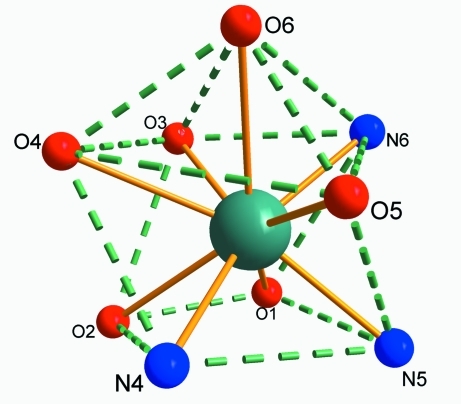
The coordination environment of the PrIII.

**Fig. 3.**
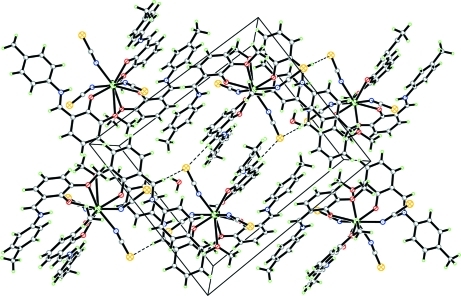
The stacking plot of the title compound, showing H-bond interactions (dashed lines) and π–π stacking interactions.

## supplementary crystallographic information

### Comment

Schiff base complexes have been received much attention for many years. By introducing diverse groups of different shapes and functions, Schiff bases also have potential applications in material science, biological, encapsulation, hydrometallurgy, *et al.* O-vanillin derived Schiff base complexes have been absorbed considerable attention in the past decades due to the intriguing biological activities of *o*-vanillin and the convenience in Schiff bases synthesis. Interested in this field, we have been synthesized several analogous Schiff bases derived from *o*-vanillin and prepared their transitional and rare metal complexes further. In a few of articles we have reported our partial research results (Zhao *et al.*, 2007; Zhu *et al.*, 2005; Xian *et al.*, 2008; Li *et al.*, 2008). Herein, we describe a new Pr^III^ complex.

The structure of complex (1) was shown in Fig.1 and the coordination environment of Pr^III^ was shown in Fig. 2. In this complex the Pr^III^ is nine-coordinated by three nitrogen atoms from three thiocyanate ions and six O atoms from the Schiff base 2-methoxy-6-[(4-methylphenyl)iminomethyl]phenol (H*L*), which can be described as a distorted monocapped square antiprism. H*L* ligands coordinate to the Pr^III^ ion with bidentate-chelate mode using oxygen atom from deprotonated phenolic hydroxyl groups and methoxyl groups. The Pr—O and Pr—N bond distances were listed in Table 1, The distances between Pr^III^ and methoxyl O atoms are obvious longer than Pr—O(phenolic) bond distances, which are similar to the analogous complexes (Zhao *et al.*, 2007; Li *et al.*, 2008, Liu *et al.*, 2009). The thiocyanate anions coordinate the Pr^III^ with N terminal with distances from 2.515–2.562 Å.

The hydrogen bonds and π–π weak non-covalent interactions lend stability to the structure. The stacking plot of this compound was shown in Fig. 3. In the structure, In H*L* ligand, three protons of phenolic hydroxyl groups considered to have transferred to imine N atoms involve in forming intramolecular hydrogen bonds. There are no classic hydrogen bonds between the adjacent molecules, but exist C—H···S weak hydrogen bonds. The π–π interactions exist both intra and extra molecules between the approximate paralleled participating benzene rings, which may be the primary forces keep the complex molecules packing together.

### Experimental

Reagents and solvents used were of commercially available quality and without purified before using. The Schiff base ligand 2-methoxy-6-[(4-methylphenyl)iminomethyl]phenol (H*L*) was synthesized from condensation of *o*-vanillin and *p*-methylaniline. The title compound was synthesized by traditional method. 3 mmol H*L* ligand was dissolved in methanol, then 1 mmol Pr(NO_3_)_3_ (in methanol) was added to the upper solution. The mixture solution was stirred for 2 h at room temperature. Furthermore, 3 mmol NH_4_SCN (dissolved in methanol) was added. The mixture was stirred again for 8 h at room temperature. At last, deposit was filtered out and the reddish-brown solution was kept in the open air. The red crystal was obtained after several days.

### Refinement

The structure was solved by direct methods and successive Fourier difference synthesis. The H atoms bonded to C and N atoms were positioned geometrically and refined using a riding model [aliphatic C—H =0.96 Å (*U*_iso_(H) = 1.5*U*_eq_(C)), aromatic C—H = 0.93 Å (*U*_iso_(H) = 1.2*U*_eq_(C)) and N—H = 0.86 Å, *U*_iso_(H) = 1.2*U*_eq_(C)]. The H atoms coordinated water molecule were located in a difference Fourier maps and refined with O—H distance restraints of 0.85 (2) and *U*_iso_(H) = 1.5*U*_eq_(O).

### Crystal data

**Table:**

[Pr(NCS)_3_(C_15_H_15_NO_2_)_3_]·H_2_O	*F*(000) = 2160
*M**_r_* = 1057.01	*D*_x_ = 1.384 Mg m^−^^3^
Monoclinic, *P*2_1_/*c*	Mo *K*α radiation, λ = 0.71073 Å
Hall symbol: -P 2ybc	Cell parameters from 4646 reflections
*a* = 16.6770 (6) Å	θ = 1.7–25.0°
*b* = 14.2420 (5) Å	µ = 1.14 mm^−^^1^
*c* = 22.2021 (8) Å	*T* = 296 K
β = 105.810 (2)°	Block, red
*V* = 5073.8 (3) Å^3^	0.18 × 0.16 × 0.04 mm
*Z* = 4	

### Data collection

**Table:**

Bruker APEXII area-detector diffractometer	8927 independent reflections
Radiation source: fine-focus sealed tube	6217 reflections with *I* > 2σ(*I*)
graphite	*R*_int_ = 0.067
φ and ω scans	θ_max_ = 25.0°, θ_min_ = 1.7°
Absorption correction: multi-scan (*SADABS*; Sheldrick, 1996)	*h* = −19→19
*T*_min_ = 0.819, *T*_max_ = 0.956	*k* = −16→16
39629 measured reflections	*l* = −26→26

### Refinement

**Table:**

Refinement on *F*^2^	Primary atom site location: structure-invariant direct methods
Least-squares matrix: full	Secondary atom site location: difference Fourier map
*R*[*F*^2^ > 2σ(*F*^2^)] = 0.039	Hydrogen site location: inferred from neighbouring sites
*wR*(*F*^2^) = 0.098	H atoms treated by a mixture of independent and constrained refinement
*S* = 1.01	*w* = 1/[σ^2^(*F*_o_^2^) + (0.0462*P*)^2^ + 0.1769*P*] where *P* = (*F*_o_^2^ + 2*F*_c_^2^)/3
8927 reflections	(Δ/σ)_max_ = 0.043
589 parameters	Δρ_max_ = 0.55 e Å^−^^3^
9 restraints	Δρ_min_ = −0.40 e Å^−^^3^

### Special details

**Table:**

Geometry. All e.s.d.'s (except the e.s.d. in the dihedral angle between two l.s. planes) are estimated using the full covariance matrix. The cell e.s.d.'s are taken into account individually in the estimation of e.s.d.'s in distances, angles and torsion angles; correlations between e.s.d.'s in cell parameters are only used when they are defined by crystal symmetry. An approximate (isotropic) treatment of cell e.s.d.'s is used for estimating e.s.d.'s involving l.s. planes.
Refinement. Refinement of *F*^2^ against ALL reflections. The weighted *R*-factor *wR* and goodness of fit *S* are based on *F*^2^, conventional *R*-factors *R* are based on *F*, with *F* set to zero for negative *F*^2^. The threshold expression of *F*^2^ > σ(*F*^2^) is used only for calculating *R*-factors(gt) *etc*. and is not relevant to the choice of reflections for refinement. *R*-factors based on *F*^2^ are statistically about twice as large as those based on *F*, and *R*- factors based on ALL data will be even larger.

### Fractional atomic coordinates and isotropic or equivalent isotropic displacement parameters (Å^2^)

**Table:**

	*x*	*y*	*z*	*U*_iso_*/*U*_eq_	
Pr	0.212161 (10)	0.224658 (13)	0.892590 (8)	0.04806 (5)	
S1	−0.05204 (9)	0.13555 (12)	0.73132 (8)	0.1423 (6)	
S2	0.25332 (6)	−0.10349 (9)	1.01053 (6)	0.0999 (4)	
S3	0.33254 (9)	0.39684 (10)	1.10377 (5)	0.1191 (5)	
N1	0.47905 (15)	0.12318 (18)	1.00203 (11)	0.0503 (7)	
H1A	0.4258	0.1270	0.9874	0.060*	
N2	0.45556 (16)	0.3989 (2)	0.93078 (12)	0.0610 (8)	
H2A	0.4104	0.3749	0.9356	0.073*	
N3	−0.02353 (15)	0.07807 (18)	0.91915 (11)	0.0509 (7)	
H3A	0.0253	0.0956	0.9178	0.061*	
N4	0.1111 (2)	0.1421 (3)	0.80336 (15)	0.0965 (12)	
N5	0.21804 (18)	0.0642 (2)	0.94577 (15)	0.0773 (10)	
N6	0.27758 (18)	0.2739 (2)	1.00482 (13)	0.0686 (9)	
O1	0.35015 (12)	0.15979 (16)	0.91093 (9)	0.0540 (6)	
O1W	0.83860 (12)	0.06713 (16)	1.23844 (9)	0.676 (14)	
H1WB	0.861 (3)	0.1157 (15)	1.259 (3)	1.014*	
H1WA	0.8812 (17)	0.031 (3)	1.247 (6)	1.014*	
O2	0.28259 (14)	0.19880 (17)	0.79334 (9)	0.0645 (7)	
O3	0.30182 (13)	0.35646 (15)	0.88812 (9)	0.0568 (6)	
O4	0.16395 (15)	0.35905 (18)	0.79935 (10)	0.0716 (8)	
O5	0.08437 (13)	0.20881 (14)	0.92029 (10)	0.0568 (6)	
O6	0.12286 (14)	0.38343 (16)	0.91669 (11)	0.0653 (7)	
C1	0.48704 (18)	0.1579 (2)	0.89903 (14)	0.0476 (8)	
C2	0.40013 (19)	0.1696 (2)	0.87554 (14)	0.0485 (8)	
C3	0.3681 (2)	0.1914 (2)	0.81113 (14)	0.0534 (9)	
C4	0.4189 (2)	0.2025 (2)	0.77316 (15)	0.0631 (10)	
H4A	0.3963	0.2163	0.7310	0.076*	
C5	0.5054 (2)	0.1931 (3)	0.79749 (17)	0.0714 (11)	
H5A	0.5401	0.2021	0.7715	0.086*	
C6	0.5392 (2)	0.1708 (3)	0.85876 (15)	0.0626 (10)	
H6A	0.5966	0.1640	0.8743	0.075*	
C7	0.52250 (19)	0.1356 (2)	0.96227 (15)	0.0525 (9)	
H7A	0.5801	0.1294	0.9764	0.063*	
C8	0.2427 (3)	0.2025 (4)	0.72728 (16)	0.0934 (15)	
H8C	0.1996	0.1559	0.7166	0.140*	
H8B	0.2831	0.1905	0.7046	0.140*	
H8A	0.2189	0.2636	0.7165	0.140*	
C9	0.50968 (19)	0.1039 (2)	1.06729 (14)	0.0503 (8)	
C10	0.5913 (2)	0.0798 (2)	1.09475 (15)	0.0602 (10)	
H10A	0.6286	0.0730	1.0706	0.072*	
C11	0.6171 (2)	0.0662 (3)	1.15818 (17)	0.0731 (12)	
H11A	0.6723	0.0496	1.1764	0.088*	
C12	0.5645 (3)	0.0761 (3)	1.19596 (17)	0.0784 (12)	
C13	0.4827 (3)	0.0982 (3)	1.16702 (18)	0.0867 (13)	
H13A	0.4451	0.1036	1.1910	0.104*	
C14	0.4550 (2)	0.1128 (3)	1.10301 (16)	0.0698 (11)	
H14A	0.3996	0.1285	1.0845	0.084*	
C15	0.5951 (3)	0.0605 (4)	1.26608 (18)	0.1164 (18)	
H15A	0.6537	0.0468	1.2774	0.175*	
H15B	0.5656	0.0087	1.2777	0.175*	
H15C	0.5855	0.1161	1.2875	0.175*	
C16	0.3713 (2)	0.4573 (3)	0.83420 (15)	0.0647 (10)	
C17	0.3005 (2)	0.4080 (2)	0.83957 (15)	0.0581 (9)	
C18	0.2269 (2)	0.4138 (3)	0.78989 (15)	0.0658 (10)	
C19	0.2230 (3)	0.4700 (3)	0.73899 (18)	0.0919 (14)	
H19A	0.1734	0.4752	0.7075	0.110*	
C20	0.2929 (3)	0.5189 (4)	0.7343 (2)	0.1121 (17)	
H20A	0.2899	0.5557	0.6991	0.134*	
C21	0.3658 (3)	0.5140 (3)	0.7802 (2)	0.0966 (14)	
H21A	0.4120	0.5476	0.7764	0.116*	
C22	0.4475 (2)	0.4495 (3)	0.88076 (17)	0.0688 (11)	
H22A	0.4937	0.4816	0.8755	0.083*	
C23	0.0823 (3)	0.3711 (3)	0.7574 (2)	0.0994 (16)	
H23C	0.0665	0.3152	0.7329	0.149*	
H23A	0.0828	0.4233	0.7300	0.149*	
H23B	0.0430	0.3831	0.7810	0.149*	
C24	0.5290 (2)	0.3785 (2)	0.97819 (17)	0.0617 (10)	
C25	0.6074 (2)	0.3848 (3)	0.9690 (2)	0.0816 (13)	
H25A	0.6140	0.4057	0.9310	0.098*	
C26	0.6757 (3)	0.3600 (3)	1.0167 (2)	0.0972 (16)	
H26A	0.7282	0.3638	1.0100	0.117*	
C27	0.6694 (3)	0.3296 (3)	1.0739 (2)	0.0918 (15)	
C28	0.5902 (3)	0.3248 (3)	1.08205 (19)	0.0828 (13)	
H28A	0.5841	0.3050	1.1205	0.099*	
C29	0.5200 (2)	0.3484 (3)	1.03516 (18)	0.0700 (11)	
H29A	0.4674	0.3440	1.0418	0.084*	
C30	0.7450 (3)	0.3013 (4)	1.1258 (3)	0.134 (2)	
H30A	0.7278	0.2828	1.1620	0.201*	
H30B	0.7826	0.3536	1.1364	0.201*	
H30C	0.7726	0.2498	1.1120	0.201*	
C31	−0.0547 (2)	0.2409 (2)	0.92143 (16)	0.0581 (9)	
C32	0.0262 (2)	0.2684 (2)	0.92020 (14)	0.0499 (8)	
C33	0.0432 (2)	0.3660 (2)	0.91852 (15)	0.0593 (10)	
C34	−0.0174 (3)	0.4306 (3)	0.91777 (19)	0.0815 (12)	
H34A	−0.0058	0.4942	0.9157	0.098*	
C35	−0.0957 (3)	0.4025 (3)	0.9200 (2)	0.1002 (15)	
H35A	−0.1360	0.4477	0.9201	0.120*	
C36	−0.1150 (2)	0.3107 (3)	0.9221 (2)	0.0858 (13)	
H36A	−0.1680	0.2932	0.9240	0.103*	
C37	−0.0748 (2)	0.1450 (2)	0.92179 (15)	0.0590 (9)	
H37A	−0.1279	0.1289	0.9241	0.071*	
C38	0.1510 (3)	0.4783 (3)	0.9233 (2)	0.0907 (13)	
H38A	0.1083	0.5177	0.9312	0.136*	
H38B	0.1632	0.4982	0.8854	0.136*	
H38C	0.2004	0.4829	0.9576	0.136*	
C39	−0.03682 (19)	−0.0199 (2)	0.91820 (14)	0.0481 (8)	
C40	−0.1077 (2)	−0.0589 (2)	0.92811 (15)	0.0583 (9)	
H40A	−0.1492	−0.0208	0.9356	0.070*	
C41	−0.1163 (2)	−0.1552 (3)	0.92669 (15)	0.0654 (10)	
H41A	−0.1645	−0.1812	0.9331	0.079*	
C42	−0.0564 (2)	−0.2142 (2)	0.91618 (15)	0.0634 (10)	
C43	0.0147 (2)	−0.1732 (3)	0.90665 (17)	0.0705 (11)	
H43A	0.0564	−0.2113	0.8994	0.085*	
C44	0.0246 (2)	−0.0768 (3)	0.90785 (15)	0.0616 (10)	
H44A	0.0728	−0.0505	0.9016	0.074*	
C45	−0.0661 (3)	−0.3192 (3)	0.9153 (2)	0.0917 (14)	
H45A	−0.1186	−0.3352	0.9226	0.138*	
H45B	−0.0216	−0.3464	0.9475	0.138*	
H45C	−0.0641	−0.3431	0.8753	0.138*	
C46	0.0431 (3)	0.1384 (3)	0.77308 (18)	0.0851 (13)	
C47	0.23374 (19)	−0.0036 (3)	0.97341 (17)	0.0634 (10)	
C48	0.2994 (2)	0.3241 (3)	1.04593 (15)	0.0606 (10)	

### Atomic displacement parameters (Å^2^)

**Table:**

	*U*^11^	*U*^22^	*U*^33^	*U*^12^	*U*^13^	*U*^23^
Pr	0.04023 (9)	0.05242 (11)	0.04853 (9)	−0.00331 (9)	0.00700 (7)	−0.00349 (9)
S1	0.0901 (9)	0.1280 (12)	0.1672 (13)	−0.0361 (8)	−0.0356 (9)	0.0146 (10)
S2	0.0681 (6)	0.0948 (8)	0.1484 (9)	0.0321 (6)	0.0494 (6)	0.0558 (7)
S3	0.1689 (11)	0.1297 (10)	0.0717 (6)	−0.0744 (9)	0.0550 (7)	−0.0385 (7)
N1	0.0439 (14)	0.0559 (17)	0.0505 (14)	0.0037 (12)	0.0118 (12)	0.0006 (13)
N2	0.0553 (16)	0.0563 (18)	0.0695 (17)	−0.0134 (14)	0.0138 (14)	−0.0052 (15)
N3	0.0420 (14)	0.0533 (16)	0.0595 (15)	−0.0020 (13)	0.0170 (12)	0.0010 (13)
N4	0.072 (2)	0.125 (3)	0.082 (2)	−0.030 (2)	0.0035 (18)	−0.030 (2)
N5	0.0625 (18)	0.068 (2)	0.109 (2)	0.0044 (16)	0.0355 (17)	0.0196 (18)
N6	0.0703 (19)	0.080 (2)	0.0535 (16)	−0.0100 (17)	0.0143 (14)	−0.0075 (16)
O1	0.0445 (11)	0.0681 (15)	0.0517 (11)	0.0025 (11)	0.0169 (10)	0.0038 (11)
O1W	0.417 (16)	0.84 (3)	0.71 (3)	0.240 (19)	0.055 (18)	−0.08 (3)
O2	0.0581 (14)	0.0866 (18)	0.0438 (12)	0.0115 (12)	0.0053 (10)	−0.0044 (12)
O3	0.0620 (13)	0.0592 (14)	0.0446 (11)	−0.0091 (11)	0.0070 (10)	0.0031 (10)
O4	0.0628 (15)	0.0871 (19)	0.0544 (13)	0.0039 (13)	−0.0019 (11)	−0.0023 (13)
O5	0.0460 (12)	0.0459 (13)	0.0803 (14)	0.0021 (10)	0.0206 (11)	0.0009 (11)
O6	0.0650 (14)	0.0465 (13)	0.0861 (15)	−0.0098 (11)	0.0235 (12)	−0.0025 (12)
C1	0.0449 (17)	0.0469 (18)	0.0513 (17)	0.0022 (14)	0.0138 (14)	−0.0014 (15)
C2	0.0491 (17)	0.0484 (19)	0.0484 (17)	0.0025 (15)	0.0141 (14)	−0.0029 (15)
C3	0.0558 (19)	0.0514 (19)	0.0510 (18)	0.0091 (16)	0.0113 (15)	−0.0025 (15)
C4	0.076 (2)	0.068 (2)	0.0471 (18)	0.0055 (19)	0.0194 (17)	0.0036 (16)
C5	0.075 (2)	0.085 (3)	0.065 (2)	0.004 (2)	0.0367 (18)	0.0041 (19)
C6	0.0455 (18)	0.082 (3)	0.066 (2)	0.0067 (18)	0.0252 (16)	0.0004 (19)
C7	0.0413 (17)	0.053 (2)	0.064 (2)	0.0025 (15)	0.0150 (15)	−0.0008 (16)
C8	0.079 (3)	0.148 (4)	0.0434 (19)	0.025 (3)	−0.0012 (19)	−0.015 (2)
C9	0.0553 (19)	0.0458 (18)	0.0510 (17)	−0.0022 (15)	0.0166 (15)	−0.0007 (15)
C10	0.057 (2)	0.064 (2)	0.0584 (19)	0.0117 (17)	0.0137 (16)	0.0112 (17)
C11	0.072 (2)	0.071 (3)	0.066 (2)	0.007 (2)	0.0019 (19)	0.011 (2)
C12	0.109 (3)	0.069 (3)	0.058 (2)	0.017 (2)	0.023 (2)	0.0123 (19)
C13	0.111 (3)	0.093 (3)	0.068 (2)	0.027 (3)	0.045 (2)	0.016 (2)
C14	0.073 (2)	0.077 (3)	0.062 (2)	0.018 (2)	0.0223 (18)	0.0130 (19)
C15	0.176 (5)	0.111 (4)	0.057 (2)	0.027 (4)	0.022 (3)	0.019 (3)
C16	0.074 (2)	0.066 (2)	0.057 (2)	−0.0043 (19)	0.0226 (18)	0.0108 (18)
C17	0.071 (2)	0.055 (2)	0.0474 (17)	0.0033 (18)	0.0154 (16)	0.0001 (16)
C18	0.073 (2)	0.069 (2)	0.0513 (19)	0.011 (2)	0.0100 (18)	0.0007 (18)
C19	0.105 (3)	0.107 (4)	0.058 (2)	0.021 (3)	0.012 (2)	0.019 (2)
C20	0.137 (4)	0.124 (4)	0.077 (3)	0.017 (3)	0.032 (3)	0.048 (3)
C21	0.124 (3)	0.087 (3)	0.091 (3)	0.004 (3)	0.049 (3)	0.029 (2)
C22	0.080 (2)	0.055 (2)	0.079 (2)	−0.0105 (19)	0.034 (2)	0.0011 (19)
C23	0.077 (3)	0.101 (4)	0.093 (3)	0.015 (2)	−0.022 (2)	−0.002 (3)
C24	0.062 (2)	0.0446 (19)	0.075 (2)	−0.0155 (17)	0.0123 (18)	−0.0109 (17)
C25	0.063 (2)	0.079 (3)	0.103 (3)	−0.020 (2)	0.022 (2)	0.005 (2)
C26	0.053 (2)	0.089 (3)	0.138 (4)	−0.021 (2)	0.008 (3)	−0.007 (3)
C27	0.068 (3)	0.069 (3)	0.115 (3)	−0.013 (2)	−0.015 (2)	−0.011 (3)
C28	0.095 (3)	0.060 (2)	0.082 (3)	−0.011 (2)	0.004 (2)	−0.003 (2)
C29	0.063 (2)	0.059 (2)	0.084 (3)	−0.0101 (19)	0.012 (2)	−0.006 (2)
C30	0.090 (3)	0.117 (4)	0.156 (5)	−0.012 (3)	−0.031 (3)	0.005 (4)
C31	0.0517 (19)	0.055 (2)	0.070 (2)	0.0084 (16)	0.0207 (16)	0.0052 (17)
C32	0.0500 (18)	0.0463 (18)	0.0528 (17)	0.0045 (16)	0.0129 (14)	0.0030 (16)
C33	0.069 (2)	0.046 (2)	0.065 (2)	−0.0025 (18)	0.0207 (17)	0.0036 (16)
C34	0.088 (3)	0.048 (2)	0.114 (3)	0.012 (2)	0.038 (2)	−0.001 (2)
C35	0.093 (3)	0.067 (3)	0.151 (4)	0.033 (2)	0.051 (3)	0.007 (3)
C36	0.061 (2)	0.072 (3)	0.134 (3)	0.014 (2)	0.043 (2)	0.000 (3)
C37	0.0483 (18)	0.060 (2)	0.074 (2)	0.0020 (17)	0.0248 (16)	0.0042 (18)
C38	0.112 (3)	0.053 (2)	0.117 (3)	−0.022 (2)	0.046 (3)	−0.015 (2)
C39	0.0469 (17)	0.0478 (18)	0.0482 (17)	−0.0041 (15)	0.0107 (14)	−0.0002 (15)
C40	0.062 (2)	0.057 (2)	0.0601 (19)	−0.0042 (17)	0.0231 (16)	0.0003 (17)
C41	0.075 (2)	0.062 (2)	0.060 (2)	−0.0146 (19)	0.0194 (18)	0.0018 (18)
C42	0.085 (3)	0.051 (2)	0.0443 (17)	0.004 (2)	0.0014 (17)	0.0044 (16)
C43	0.065 (2)	0.058 (2)	0.083 (2)	0.009 (2)	0.0117 (19)	−0.005 (2)
C44	0.054 (2)	0.061 (2)	0.064 (2)	−0.0017 (18)	0.0073 (17)	−0.0072 (18)
C45	0.120 (3)	0.057 (2)	0.088 (3)	−0.003 (3)	0.013 (3)	0.009 (2)
C46	0.082 (3)	0.097 (3)	0.068 (2)	−0.031 (2)	0.007 (2)	−0.012 (2)
C47	0.0391 (17)	0.074 (3)	0.081 (2)	0.0064 (17)	0.0235 (17)	0.007 (2)
C48	0.065 (2)	0.072 (2)	0.0493 (18)	−0.0131 (19)	0.0227 (16)	0.0090 (18)

### Geometric parameters (Å, °)

**Table:**

Pr—O5	2.384 (2)	C15—H15A	0.9600
Pr—O1	2.409 (2)	C15—H15B	0.9600
Pr—O3	2.419 (2)	C15—H15C	0.9600
Pr—N4	2.515 (3)	C16—C17	1.406 (5)
Pr—N6	2.532 (3)	C16—C22	1.406 (5)
Pr—N5	2.562 (3)	C16—C21	1.428 (5)
Pr—O4	2.773 (2)	C17—C18	1.412 (4)
Pr—O2	2.790 (2)	C18—C19	1.372 (5)
Pr—O6	2.838 (2)	C19—C20	1.386 (6)
S1—C46	1.605 (4)	C19—H19A	0.9300
S2—C47	1.632 (4)	C20—C21	1.358 (6)
S3—C48	1.625 (4)	C20—H20A	0.9300
N1—C7	1.298 (4)	C21—H21A	0.9300
N1—C9	1.426 (4)	C22—H22A	0.9300
N1—H1A	0.8600	C23—H23C	0.9600
N2—C22	1.299 (4)	C23—H23A	0.9600
N2—C24	1.411 (4)	C23—H23B	0.9600
N2—H2A	0.8600	C24—C25	1.380 (5)
N3—C37	1.292 (4)	C24—C29	1.382 (5)
N3—C39	1.412 (4)	C25—C26	1.373 (5)
N3—H3A	0.8600	C25—H25A	0.9300
N4—C46	1.152 (5)	C26—C27	1.372 (6)
N5—C47	1.135 (4)	C26—H26A	0.9300
N6—C48	1.138 (4)	C27—C28	1.382 (6)
O1—C2	1.299 (4)	C27—C30	1.513 (6)
O1W—H1WB	0.86000	C28—C29	1.380 (5)
O1W—H1WA	0.86000	C28—H28A	0.9300
O2—C3	1.376 (4)	C29—H29A	0.9300
O2—C8	1.437 (4)	C30—H30A	0.9600
O3—C17	1.299 (4)	C30—H30B	0.9600
O4—C18	1.368 (4)	C30—H30C	0.9600
O4—C23	1.436 (4)	C31—C37	1.407 (5)
O5—C32	1.289 (4)	C31—C32	1.412 (5)
O6—C33	1.364 (4)	C31—C36	1.417 (5)
O6—C38	1.425 (4)	C32—C33	1.421 (4)
C1—C7	1.403 (4)	C33—C34	1.363 (5)
C1—C2	1.410 (4)	C34—C35	1.380 (6)
C1—C6	1.419 (4)	C34—H34A	0.9300
C2—C3	1.418 (4)	C35—C36	1.351 (6)
C3—C4	1.357 (5)	C35—H35A	0.9300
C4—C5	1.403 (5)	C36—H36A	0.9300
C4—H4A	0.9300	C37—H37A	0.9300
C5—C6	1.361 (5)	C38—H38A	0.9600
C5—H5A	0.9300	C38—H38B	0.9600
C6—H6A	0.9300	C38—H38C	0.9600
C7—H7A	0.9300	C39—C44	1.373 (5)
C8—H8C	0.9600	C39—C40	1.377 (4)
C8—H8B	0.9600	C40—C41	1.378 (5)
C8—H8A	0.9600	C40—H40A	0.9300
C9—C14	1.368 (5)	C41—C42	1.374 (5)
C9—C10	1.375 (4)	C41—H41A	0.9300
C10—C11	1.370 (5)	C42—C43	1.390 (5)
C10—H10A	0.9300	C42—C45	1.503 (5)
C11—C12	1.376 (5)	C43—C44	1.383 (5)
C11—H11A	0.9300	C43—H43A	0.9300
C12—C13	1.377 (5)	C44—H44A	0.9300
C12—C15	1.517 (5)	C45—H45A	0.9600
C13—C14	1.385 (5)	C45—H45B	0.9600
C13—H13A	0.9300	C45—H45C	0.9600
C14—H14A	0.9300		
			
O5—Pr—O1	143.11 (7)	C12—C15—H15C	109.5
O5—Pr—O3	133.63 (7)	H15A—C15—H15C	109.5
O1—Pr—O3	74.44 (7)	H15B—C15—H15C	109.5
O5—Pr—N4	72.84 (10)	C17—C16—C22	120.6 (3)
O1—Pr—N4	110.96 (10)	C17—C16—C21	119.3 (3)
O3—Pr—N4	128.27 (10)	C22—C16—C21	120.0 (4)
O5—Pr—N6	87.03 (9)	O3—C17—C16	121.8 (3)
O1—Pr—N6	78.71 (9)	O3—C17—C18	119.7 (3)
O3—Pr—N6	73.74 (8)	C16—C17—C18	118.5 (3)
N4—Pr—N6	157.10 (11)	O4—C18—C19	126.4 (3)
O5—Pr—N5	73.82 (8)	O4—C18—C17	112.8 (3)
O1—Pr—N5	70.44 (8)	C19—C18—C17	120.8 (4)
O3—Pr—N5	139.89 (8)	C18—C19—C20	120.3 (4)
N4—Pr—N5	83.07 (12)	C18—C19—H19A	119.9
N6—Pr—N5	80.87 (10)	C20—C19—H19A	119.9
O5—Pr—O4	99.22 (7)	C21—C20—C19	121.1 (4)
O1—Pr—O4	117.40 (7)	C21—C20—H20A	119.4
O3—Pr—O4	59.36 (7)	C19—C20—H20A	119.4
N4—Pr—O4	74.87 (10)	C20—C21—C16	119.9 (4)
N6—Pr—O4	120.21 (9)	C20—C21—H21A	120.0
N5—Pr—O4	157.94 (9)	C16—C21—H21A	120.0
O5—Pr—O2	142.14 (7)	N2—C22—C16	122.3 (3)
O1—Pr—O2	59.64 (6)	N2—C22—H22A	118.8
O3—Pr—O2	70.85 (7)	C16—C22—H22A	118.8
N4—Pr—O2	69.63 (9)	O4—C23—H23C	109.5
N6—Pr—O2	130.75 (8)	O4—C23—H23A	109.5
N5—Pr—O2	105.95 (9)	H23C—C23—H23A	109.5
O4—Pr—O2	66.44 (7)	O4—C23—H23B	109.5
O5—Pr—O6	58.33 (7)	H23C—C23—H23B	109.5
O1—Pr—O6	143.12 (7)	H23A—C23—H23B	109.5
O3—Pr—O6	75.60 (7)	C25—C24—C29	119.9 (3)
N4—Pr—O6	104.35 (10)	C25—C24—N2	122.8 (3)
N6—Pr—O6	72.62 (9)	C29—C24—N2	117.2 (3)
N5—Pr—O6	125.46 (9)	C26—C25—C24	119.3 (4)
O4—Pr—O6	61.99 (7)	C26—C25—H25A	120.4
O2—Pr—O6	127.57 (7)	C24—C25—H25A	120.4
C7—N1—C9	127.3 (3)	C27—C26—C25	122.6 (4)
C7—N1—H1A	116.4	C27—C26—H26A	118.7
C9—N1—H1A	116.4	C25—C26—H26A	118.7
C22—N2—C24	128.2 (3)	C26—C27—C28	117.0 (4)
C22—N2—H2A	115.9	C26—C27—C30	122.0 (5)
C24—N2—H2A	115.9	C28—C27—C30	121.0 (5)
C37—N3—C39	128.8 (3)	C29—C28—C27	122.3 (4)
C37—N3—H3A	115.6	C29—C28—H28A	118.9
C39—N3—H3A	115.6	C27—C28—H28A	118.9
C46—N4—Pr	145.4 (4)	C28—C29—C24	118.9 (4)
C47—N5—Pr	169.0 (3)	C28—C29—H29A	120.5
C48—N6—Pr	157.1 (3)	C24—C29—H29A	120.5
C2—O1—Pr	126.68 (18)	C27—C30—H30A	109.5
H1WB—O1W—H1WA	99.00	C27—C30—H30B	109.5
C3—O2—C8	116.8 (3)	H30A—C30—H30B	109.5
C3—O2—Pr	114.19 (17)	C27—C30—H30C	109.5
C8—O2—Pr	128.6 (2)	H30A—C30—H30C	109.5
C17—O3—Pr	126.99 (19)	H30B—C30—H30C	109.5
C18—O4—C23	117.6 (3)	C37—C31—C32	120.0 (3)
C18—O4—Pr	115.42 (19)	C37—C31—C36	120.7 (3)
C23—O4—Pr	126.8 (2)	C32—C31—C36	119.4 (3)
C32—O5—Pr	131.5 (2)	O5—C32—C31	122.7 (3)
C33—O6—C38	117.8 (3)	O5—C32—C33	119.3 (3)
C33—O6—Pr	115.25 (19)	C31—C32—C33	118.0 (3)
C38—O6—Pr	126.9 (2)	C34—C33—O6	127.0 (3)
C7—C1—C2	120.5 (3)	C34—C33—C32	120.5 (3)
C7—C1—C6	119.7 (3)	O6—C33—C32	112.4 (3)
C2—C1—C6	119.8 (3)	C33—C34—C35	120.6 (4)
O1—C2—C1	121.8 (3)	C33—C34—H34A	119.7
O1—C2—C3	120.4 (3)	C35—C34—H34A	119.7
C1—C2—C3	117.8 (3)	C36—C35—C34	121.2 (4)
C4—C3—O2	125.9 (3)	C36—C35—H35A	119.4
C4—C3—C2	121.7 (3)	C34—C35—H35A	119.4
O2—C3—C2	112.5 (3)	C35—C36—C31	120.2 (4)
C3—C4—C5	120.0 (3)	C35—C36—H36A	119.9
C3—C4—H4A	120.0	C31—C36—H36A	119.9
C5—C4—H4A	120.0	N3—C37—C31	123.7 (3)
C6—C5—C4	120.6 (3)	N3—C37—H37A	118.2
C6—C5—H5A	119.7	C31—C37—H37A	118.2
C4—C5—H5A	119.7	O6—C38—H38A	109.5
C5—C6—C1	120.2 (3)	O6—C38—H38B	109.5
C5—C6—H6A	119.9	H38A—C38—H38B	109.5
C1—C6—H6A	119.9	O6—C38—H38C	109.5
N1—C7—C1	123.4 (3)	H38A—C38—H38C	109.5
N1—C7—H7A	118.3	H38B—C38—H38C	109.5
C1—C7—H7A	118.3	C44—C39—C40	120.0 (3)
O2—C8—H8C	109.5	C44—C39—N3	117.7 (3)
O2—C8—H8B	109.5	C40—C39—N3	122.3 (3)
H8C—C8—H8B	109.5	C39—C40—C41	119.1 (3)
O2—C8—H8A	109.5	C39—C40—H40A	120.5
H8C—C8—H8A	109.5	C41—C40—H40A	120.5
H8B—C8—H8A	109.5	C42—C41—C40	122.5 (3)
C14—C9—C10	120.1 (3)	C42—C41—H41A	118.8
C14—C9—N1	117.4 (3)	C40—C41—H41A	118.8
C10—C9—N1	122.4 (3)	C41—C42—C43	117.3 (3)
C11—C10—C9	119.1 (3)	C41—C42—C45	122.1 (4)
C11—C10—H10A	120.4	C43—C42—C45	120.6 (4)
C9—C10—H10A	120.4	C44—C43—C42	121.1 (4)
C10—C11—C12	122.6 (4)	C44—C43—H43A	119.4
C10—C11—H11A	118.7	C42—C43—H43A	119.4
C12—C11—H11A	118.7	C39—C44—C43	119.9 (3)
C11—C12—C13	117.0 (3)	C39—C44—H44A	120.0
C11—C12—C15	121.2 (4)	C43—C44—H44A	120.0
C13—C12—C15	121.8 (4)	C42—C45—H45A	109.5
C12—C13—C14	121.6 (4)	C42—C45—H45B	109.5
C12—C13—H13A	119.2	H45A—C45—H45B	109.5
C14—C13—H13A	119.2	C42—C45—H45C	109.5
C9—C14—C13	119.5 (3)	H45A—C45—H45C	109.5
C9—C14—H14A	120.3	H45B—C45—H45C	109.5
C13—C14—H14A	120.3	N4—C46—S1	178.8 (5)
C12—C15—H15A	109.5	N5—C47—S2	177.4 (4)
C12—C15—H15B	109.5	N6—C48—S3	178.4 (3)
H15A—C15—H15B	109.5		
			
O5—Pr—N4—C46	39.5 (6)	C6—C1—C2—C3	1.8 (5)
O1—Pr—N4—C46	−179.4 (6)	C8—O2—C3—C4	11.4 (5)
O3—Pr—N4—C46	−93.0 (6)	Pr—O2—C3—C4	−162.1 (3)
N6—Pr—N4—C46	69.0 (7)	C8—O2—C3—C2	−168.2 (3)
N5—Pr—N4—C46	114.7 (6)	Pr—O2—C3—C2	18.3 (3)
O4—Pr—N4—C46	−65.4 (6)	O1—C2—C3—C4	179.6 (3)
O2—Pr—N4—C46	−135.4 (6)	C1—C2—C3—C4	−1.1 (5)
O6—Pr—N4—C46	−10.2 (6)	O1—C2—C3—O2	−0.7 (4)
O5—Pr—N5—C47	−142.0 (17)	C1—C2—C3—O2	178.5 (3)
O1—Pr—N5—C47	28.7 (16)	O2—C3—C4—C5	179.9 (3)
O3—Pr—N5—C47	−1.5 (17)	C2—C3—C4—C5	−0.5 (5)
N4—Pr—N5—C47	143.9 (17)	C3—C4—C5—C6	1.5 (6)
N6—Pr—N5—C47	−52.4 (17)	C4—C5—C6—C1	−0.8 (6)
O4—Pr—N5—C47	143.8 (16)	C7—C1—C6—C5	−179.6 (3)
O2—Pr—N5—C47	77.5 (17)	C2—C1—C6—C5	−0.9 (5)
O6—Pr—N5—C47	−113.4 (17)	C9—N1—C7—C1	−177.5 (3)
O5—Pr—N6—C48	−91.9 (7)	C2—C1—C7—N1	1.0 (5)
O1—Pr—N6—C48	122.3 (7)	C6—C1—C7—N1	179.7 (3)
O3—Pr—N6—C48	45.4 (7)	C7—N1—C9—C14	166.5 (3)
N4—Pr—N6—C48	−120.0 (7)	C7—N1—C9—C10	−11.5 (5)
N5—Pr—N6—C48	−166.0 (7)	C14—C9—C10—C11	−0.8 (5)
O4—Pr—N6—C48	7.0 (8)	N1—C9—C10—C11	177.2 (3)
O2—Pr—N6—C48	90.8 (7)	C9—C10—C11—C12	−0.4 (6)
O6—Pr—N6—C48	−34.2 (7)	C10—C11—C12—C13	1.7 (6)
O5—Pr—O1—C2	160.6 (2)	C10—C11—C12—C15	−179.9 (4)
O3—Pr—O1—C2	−54.1 (2)	C11—C12—C13—C14	−1.9 (6)
N4—Pr—O1—C2	71.5 (3)	C15—C12—C13—C14	179.7 (4)
N6—Pr—O1—C2	−130.1 (2)	C10—C9—C14—C13	0.5 (6)
N5—Pr—O1—C2	145.6 (3)	N1—C9—C14—C13	−177.6 (3)
O4—Pr—O1—C2	−11.8 (3)	C12—C13—C14—C9	0.9 (6)
O2—Pr—O1—C2	22.5 (2)	Pr—O3—C17—C16	−155.2 (3)
O6—Pr—O1—C2	−90.8 (2)	Pr—O3—C17—C18	23.4 (4)
O5—Pr—O2—C3	−159.49 (19)	C22—C16—C17—O3	2.3 (5)
O1—Pr—O2—C3	−20.3 (2)	C21—C16—C17—O3	−179.0 (3)
O3—Pr—O2—C3	62.5 (2)	C22—C16—C17—C18	−176.3 (3)
N4—Pr—O2—C3	−151.6 (2)	C21—C16—C17—C18	2.4 (5)
N6—Pr—O2—C3	16.1 (3)	C23—O4—C18—C19	−10.0 (6)
N5—Pr—O2—C3	−75.5 (2)	Pr—O4—C18—C19	164.9 (3)
O4—Pr—O2—C3	126.6 (2)	C23—O4—C18—C17	169.8 (3)
O6—Pr—O2—C3	115.7 (2)	Pr—O4—C18—C17	−15.3 (4)
O5—Pr—O2—C8	28.0 (4)	O3—C17—C18—O4	−1.8 (5)
O1—Pr—O2—C8	167.1 (3)	C16—C17—C18—O4	176.8 (3)
O3—Pr—O2—C8	−110.1 (3)	O3—C17—C18—C19	178.0 (3)
N4—Pr—O2—C8	35.8 (3)	C16—C17—C18—C19	−3.4 (6)
N6—Pr—O2—C8	−156.4 (3)	O4—C18—C19—C20	−177.3 (4)
N5—Pr—O2—C8	112.0 (3)	C17—C18—C19—C20	2.9 (6)
O4—Pr—O2—C8	−46.0 (3)	C18—C19—C20—C21	−1.4 (8)
O6—Pr—O2—C8	−56.9 (3)	C19—C20—C21—C16	0.4 (8)
O5—Pr—O3—C17	−93.8 (3)	C17—C16—C21—C20	−0.9 (6)
O1—Pr—O3—C17	114.4 (3)	C22—C16—C21—C20	177.8 (4)
N4—Pr—O3—C17	9.7 (3)	C24—N2—C22—C16	174.1 (3)
N6—Pr—O3—C17	−163.1 (3)	C17—C16—C22—N2	−1.5 (6)
N5—Pr—O3—C17	143.9 (3)	C21—C16—C22—N2	179.8 (4)
O4—Pr—O3—C17	−21.7 (2)	C22—N2—C24—C25	−21.2 (6)
O2—Pr—O3—C17	51.7 (2)	C22—N2—C24—C29	160.7 (4)
O6—Pr—O3—C17	−87.4 (3)	C29—C24—C25—C26	0.7 (6)
O5—Pr—O4—C18	154.0 (2)	N2—C24—C25—C26	−177.3 (4)
O1—Pr—O4—C18	−30.6 (2)	C24—C25—C26—C27	−0.6 (7)
O3—Pr—O4—C18	18.3 (2)	C25—C26—C27—C28	0.0 (7)
N4—Pr—O4—C18	−136.7 (2)	C25—C26—C27—C30	179.2 (4)
N6—Pr—O4—C18	62.1 (2)	C26—C27—C28—C29	0.5 (6)
N5—Pr—O4—C18	−136.5 (3)	C30—C27—C28—C29	−178.7 (4)
O2—Pr—O4—C18	−62.7 (2)	C27—C28—C29—C24	−0.5 (6)
O6—Pr—O4—C18	107.6 (2)	C25—C24—C29—C28	−0.2 (5)
O5—Pr—O4—C23	−31.7 (3)	N2—C24—C29—C28	178.0 (3)
O1—Pr—O4—C23	143.7 (3)	Pr—O5—C32—C31	161.9 (2)
O3—Pr—O4—C23	−167.4 (3)	Pr—O5—C32—C33	−17.8 (4)
N4—Pr—O4—C23	37.6 (3)	C37—C31—C32—O5	−1.0 (5)
N6—Pr—O4—C23	−123.6 (3)	C36—C31—C32—O5	179.1 (3)
N5—Pr—O4—C23	37.8 (4)	C37—C31—C32—C33	178.7 (3)
O2—Pr—O4—C23	111.7 (3)	C36—C31—C32—C33	−1.2 (5)
O6—Pr—O4—C23	−78.1 (3)	C38—O6—C33—C34	9.4 (5)
O1—Pr—O5—C32	154.8 (2)	Pr—O6—C33—C34	−167.1 (3)
O3—Pr—O5—C32	24.1 (3)	C38—O6—C33—C32	−171.8 (3)
N4—Pr—O5—C32	−102.9 (3)	Pr—O6—C33—C32	11.7 (3)
N6—Pr—O5—C32	88.1 (3)	O5—C32—C33—C34	179.4 (3)
N5—Pr—O5—C32	169.5 (3)	C31—C32—C33—C34	−0.4 (5)
O4—Pr—O5—C32	−32.0 (3)	O5—C32—C33—O6	0.5 (4)
O2—Pr—O5—C32	−95.2 (3)	C31—C32—C33—O6	−179.3 (3)
O6—Pr—O5—C32	16.8 (2)	O6—C33—C34—C35	−179.8 (4)
O5—Pr—O6—C33	−13.66 (19)	C32—C33—C34—C35	1.5 (6)
O1—Pr—O6—C33	−151.71 (19)	C33—C34—C35—C36	−1.0 (7)
O3—Pr—O6—C33	171.8 (2)	C34—C35—C36—C31	−0.6 (7)
N4—Pr—O6—C33	45.3 (2)	C37—C31—C36—C35	−178.2 (4)
N6—Pr—O6—C33	−111.1 (2)	C32—C31—C36—C35	1.7 (6)
N5—Pr—O6—C33	−46.4 (2)	C39—N3—C37—C31	−178.9 (3)
O4—Pr—O6—C33	109.1 (2)	C32—C31—C37—N3	−2.2 (5)
O2—Pr—O6—C33	120.4 (2)	C36—C31—C37—N3	177.7 (3)
O5—Pr—O6—C38	170.2 (3)	C37—N3—C39—C44	172.5 (3)
O1—Pr—O6—C38	32.2 (3)	C37—N3—C39—C40	−8.4 (5)
O3—Pr—O6—C38	−4.4 (3)	C44—C39—C40—C41	−0.8 (5)
N4—Pr—O6—C38	−130.8 (3)	N3—C39—C40—C41	−179.8 (3)
N6—Pr—O6—C38	72.8 (3)	C39—C40—C41—C42	0.5 (5)
N5—Pr—O6—C38	137.5 (3)	C40—C41—C42—C43	−0.1 (5)
O4—Pr—O6—C38	−67.0 (3)	C40—C41—C42—C45	179.4 (3)
O2—Pr—O6—C38	−55.7 (3)	C41—C42—C43—C44	0.1 (5)
Pr—O1—C2—C1	157.8 (2)	C45—C42—C43—C44	−179.5 (3)
Pr—O1—C2—C3	−23.0 (4)	C40—C39—C44—C43	0.7 (5)
C7—C1—C2—O1	−0.3 (5)	N3—C39—C44—C43	179.8 (3)
C6—C1—C2—O1	−179.0 (3)	C42—C43—C44—C39	−0.4 (5)
C7—C1—C2—C3	−179.5 (3)		

### Hydrogen-bond geometry (Å, °)

**Table:**

*D*—H···*A*	*D*—H	H···*A*	*D*···*A*	*D*—H···*A*
N1—H1A···O1	0.86	1.88	2.572 (3)	137
N2—H2A···O3	0.86	1.85	2.552 (3)	138
N3—H3A···O5	0.86	1.88	2.585 (3)	138
